# Population growth is limited by nutritional impacts on pregnancy success in endangered Southern Resident killer whales (*Orcinus orca*)

**DOI:** 10.1371/journal.pone.0179824

**Published:** 2017-06-29

**Authors:** Samuel K. Wasser, Jessica I. Lundin, Katherine Ayres, Elizabeth Seely, Deborah Giles, Kenneth Balcomb, Jennifer Hempelmann, Kim Parsons, Rebecca Booth

**Affiliations:** 1 Center for Conservation Biology, Department of Biology, University of Washington, Seattle, WA, United States of America; 2 Center for Whale Research, Friday Harbor, Washington, United States of America; 3 Northwest Fisheries Center, National Oceanic and Atmospheric Administration Fisheries, Seattle, WA, United States of America; Pacific Northwest National Laboratory, UNITED STATES

## Abstract

The Southern Resident killer whale population (*Orcinus orca*) was listed as endangered in 2005 and shows little sign of recovery. These fish eating whales feed primarily on endangered Chinook salmon. Population growth is constrained by low offspring production for the number of reproductive females in the population. Lack of prey, increased toxins and vessel disturbance have been listed as potential causes of the whale’s decline, but partitioning these pressures has been difficult. We validated and applied temporal measures of progesterone and testosterone metabolites to assess occurrence, stage and health of pregnancy from genotyped killer whale feces collected using detection dogs. Thyroid and glucocorticoid hormone metabolites were measured from these same samples to assess physiological stress. These methods enabled us to assess pregnancy occurrence and failure as well as how pregnancy success was temporally impacted by nutritional and other stressors, between 2008 and 2014. Up to 69% of all detectable pregnancies were unsuccessful; of these, up to 33% failed relatively late in gestation or immediately post-partum, when the cost is especially high. Low availability of Chinook salmon appears to be an important stressor among these fish-eating whales as well as a significant cause of late pregnancy failure, including unobserved perinatal loss. However, release of lipophilic toxicants during fat metabolism in the nutritionally deprived animals may also provide a contributor to these cumulative effects. Results point to the importance of promoting Chinook salmon recovery to enhance population growth of Southern Resident killer whales. The physiological measures used in this study can also be used to monitor the success of actions aimed at promoting adaptive management of this important apex predator to the Pacific Northwest.

## 1. Introduction

The Southern Resident killer whales (SRKW; *Orcinus orca*) represent the southern population of the fish-eating ecotype inhabiting the northeast Pacific Ocean [1]. From late May through October, the three SRKW pods, termed J, K and L, frequent the inshore waters of Washington State and British Columbia, commonly known as the Salish Sea. Following a near 20% decline in their population during the late ‘90’s, the population was listed as endangered under the Canadian Species at Risk Act in 2001 [2] and the U.S. Endangered Species Act in 2005 [1]. Only 78 individuals (J pod = 24 individuals; K pod = 19 individuals; L pod = 35 individuals) remain in the current population as of December, 2016 [3]. Reduced availability of their preferred prey, threatened and endangered Chinook salmon, appears to be at the core of the SRKW decline [4–7], although exposure to toxicants [8], and pressure from vessel disturbance may also contribute to these cumulative effects [9].

Reduced fecundity appears to be a particularly important contributor to the SRKWs failure to recover [4]. The rate of successful pregnancy in the wild population is unknown since, to date, pregnancy is only confirmed by observation of a newborn calf. SRKW typically give birth every 5.3 years [10]. However, holding age structure and survivorship constant, fecundity rates of SRKW (0.21) are significantly lower than those of Northern Resident (0.26;) [11] or Southeast Alaskan Resident killer whales (0.27) [12], neither of which are listed as at risk. Assuming a median peak fecundity rate of 0.21, the 31 potentially reproductive females in the SRKW population should have had 48 births between 2008–2015. Yet, only 28 births were recorded during that period. The 7 adult females in K pod have not had a birth since 2011, and just two births since 2007. The 24 females in the remaining two pods (J and L) have averaged < 1 birth per pod since 2011, with no births in 2013, but had 7 births in 2015. One of the two offspring born in 2014 died [3]. This study addresses causes of the low reproductive rate in SRKWs in an effort to recommend management decisions that can enhance population growth and long-term sustainability of this endangered population.

We examine determinants of pregnancy success and failure in the SRKWs from 2008 through 2014 based on hormone measures of pregnancy occurrence and health as well as physiological stress from genotyped feces. SRKW fecal samples are located with high efficiency by specially trained detection dogs, with detection rates over five times that by trained human observers [5,13,14]. Progesterone and testosterone collectively provide reliable indices of pregnancy occurrence, timing and health in killer whales. Concentrations of both P4 and T increase several-fold during gestation, although the increase is more gradual for T. Both hormones sharply decline to pre-conception levels around parturition [15,16]. We develop and validate a noninvasive endocrine measure of pregnancy occurrence and loss in the killer whales using metabolites of progesterone (P4) and testosterone (T) excreted in their feces.

Fecal glucocorticoid (GC) and thyroid (T3) hormone metabolite measures are used to monitor nutritional and disturbance stress within and between years. These two endocrine systems work closely together to regulate energy availability and utilization to meet nutritional, growth and thermoregulatory demands [17]. GCs rapidly rise in response to poor nutrition, cold temperature and disturbance stressors, mobilizing glucose to provide energy to deal with the immediate emergency [18,19]. GC concentrations over time are particularly informative for distinguishing nutritional from boat stress since abundances of both Chinook and whale-watching boats have very similar temporal patterns. Chinook and boat abundance are both relatively low in spring, peak in mid- to late August and then decline. Yet, the GC signal from nutritional stress should be lowest when fish abundance is at its peak while highest when boat density is at its peak [5].

Thyroid hormone (triiodothyronine, T3), on the other hand, produces a more conservative response to nutritional and thermal stress, functioning by adjusting metabolism. It is also important to promote fetal brain growth during gestation [20]. While T4 is the most abundant thyroid hormone, it is directly converted to T3, which has many times the biological activity of T4 [20,21]. T3 levels are relatively slow to change when food shortages are first encountered, allowing the body to use all available fuel to search for food. If poor food conditions persist, T3 abruptly declines, lowering metabolism to prevent the body from exhausting its remaining fuel stores [21–24]. T3 may also be blunted under good food conditions when a low metabolism is needed to increase growth (e.g., to accumulate blubber stores in fall, in preparation for the relatively lean winter; [20]). In dolphins, T3 is lower in failed versus successful pregnancies at all stages of gestation [25]. T3 is relatively unresponsive to disturbance stress.

This study uses temporal patterns in P4 and T to predict pregnancy outcomes among the SRKWs and T3, GC and the T3/GC ratio to index the importance of nutritional and other stressors in their reproductive decline.

### 1.1 SRKW natural history

Mean reproductive maturity (age at first conception) in female SRKWs occurs at 9.8 years of age in captivity 12.1 years in the wild [10,26]. Maximum fecundity (probability of becoming pregnant in a single estrous cycle) of SRKW occurs between ages 20–22, increasing quickly during the first four years after sexual maturity, slowly declining from age 22 to 39, and then precipitously declining thereafter [4,10]. Gestation is approximately 18 months, making the prior year’s salmon availability particularly important to fecundity [11,27].

During our late May through October study period, the SRKWs primarily feed on Chinook salmon, increasingly dominated by Fraser River Chinook (FRC) returning to spawn in nearby rivers [28,29]. SRKWs generally spend the remainder of the year outside the Salish Sea, moving up and down the Pacific Coast, from CA to Southeast AK [6]. K and L pods tend to spend more time further south than does J pod in winter, while J pod frequents the Salish Sea more than does K and L pods in summer and winter. Nutritional demands on SRKW are presumed to be greatest in winter when their salmonid prey are more widely dispersed, smaller in size and other non-salmonid prey appear to be a larger fraction of the diet [6,29,30]. Thermoregulatory demands may also influence nutritional demands during winter. SRKW then transition to spring, eventually subsisting on a diminishing number of spring/summer run adult Chinook salmon approaching river mouths inside and outside the Salish Sea until the Fraser River Chinook (FRC) runs peak in mid- to late-August.

Temporal patterns in fecal GC and T3 concentrations [5], combined with radio-tagging data [28], suggest that early spring interior race Columbia River Chinook (CRC) runs are also important to SRKW nutrition. The CRC run increases from mid-March to the end of May based on estimates at the Bonneville dam [31] and have some of the highest fat content of any adult salmon to support their extremely long freshwater spawning migration [32,33]. Foraging on the fat rich Columbia River Chinook in early spring was hypothesized to replenish the killer whales after the long winter and sustain them until the temporally and quantitatively variable mid to late August peak in Fraser River Chinook (FRC) occurs (S1 Fig). T3 concentrations in fecal samples collected between 2007 and 2009 were consistently at their highest when the SRKW first arrived in the Salish Sea in late spring [5]. Presumably, this occurred because the whales arrived after feeding on the fat rich Columbia River Chinook. SRKW were detected twice as frequently at the Columbia River in early spring than expected by chance [28]. This argument is further supported by increases in serum thyroid stimulating hormone, T4 and T3 in fasting humans and rats in response to leptin injections [20]. With FRC runs still quite low, T3 levels then fell precipitously. GC concentrations when the SRKWs first arrive in the Salish Sea in late spring were also relatively high, further reflecting the comparatively low FRC abundance at that time, and consistent with the precipitous decline in T3 shortly following SRKW arrival [5].

## 2. Methods

### 2.1 Ethics statement

Fecal samples were collected in United States waters under National Marine Fisheries Service permits 532-1822-00, 532–1822, 10045 and 17344. Samples were collected in Canadian waters under Marine Mammal License numbers 2008–16, 2009–08, 2010–09 and 2012–08, as well as Species at Risk Act permits numbered 91, 102, 109 and 155. Sample collection methods were approved by the University of Washington’s Institutional Animal Care and Use Committee (IACUC) under protocol 2850–08.

### 2.2 Scat (fecal) sampling using detection dogs

Scat sampling occurred in the Salish Sea between late May and October, from 2008–2014, coinciding with the time the SRKWs frequent the study area. Whenever possible, we aimed to evenly sample each pod by starting at the front of the pod’s direction of travel, continuing to sample until the pod passes and then returning again to the front of the pod.

Scat samples are located by detection dogs trained to locate SRKW scat floating on the water’s surface [5,13,14]. The use of detection dogs greatly increases sample size due to their remarkable ability to smell SRKW scats at distances up to one nautical mile away, even in fast moving currents. The detection dog rides on the bow of the boat, driven perpendicular to the wind, beginning at least 200 yards downwind from an area where the whales have just traveled. As the boat approaches the edge of the scent cone emanating from the sample, the dog’s behavior suddenly changes from resting to actively perched far over the bow of the boat, anticipating its reward for sample detection. As the boat passes through the center of the scent cone, where the odor is strongest, the dog leans heavily over the windward side of the boat, following the strongest scent, informing the handler to direct the boat driver to turn into the wind. Subtle cues by the dog, relative to wind direction, allow the driver to stay on the scent line until the sample is reached. The dog typically becomes restless, often whining at that point because the scent surrounds the boat and thus no longer has a clear direction. If at any time the boat travels out of the scent cone, the dog changes position and looks back to where the scent was strongest. The handler then directs the driver to circle back into the scent cone until the dog’s change in behavior once again alerts the handler it has redetected the scent.

As soon as the sample is visually located, a 1-liter polypropylene beaker fastened to a 3–6 foot pole is used to scoop the sample by skimming the surface just under the sample. The first sample out of the water is presented to the dog, which is followed immediately by the toy reward and a few minutes of play. Meanwhile, the crew continues to scoop all remaining sample pieces from the water’s surface. The majority of water is carefully poured off the sample, and the sample pieces are collected into a 50 mL polypropylene tube, centrifuged, and the remaining seawater is decanted. The sample is placed on dry ice until stored frozen at -20°C that evening and remains at that temperature until processed in the lab. Fecal samples range in size from 0.5 to 300 mls, but a typical sample collection volume is 2 mls. Fortunately, the consistency of SRKW scat makes the hormones fairly evenly distributed even in small samples (Ayres and Wasser, unpublished data).

### 2.3 Fecal DNA and hormone measures

Once thawed for hormone extraction, the homogenized sample is swabbed for DNA using a synthetic tip. The swab is then kept frozen at -20°C until being genotyped for species, sex, pod, and individual identification by NOAA NW Fisheries Science Center [34]. 76% of all individuals are currently genotyped to the individual, and 88% of all adult females. Fecal hormone metabolites of glucocorticoid (GC), thyroid (triiodothyronine, T3), testosterone (T) and progesterone (P4) are extracted using methods described in [21] and measured using assays in Wasser et al. [35] (P4), [36] (GC), [21] (T3)] and Vellosa et al. [37] (T). Briefly, each sample is thawed once and centrifuged (2,200 rpm for 20 minutes), allowing any excess salt-water to be decanted. Samples are lyophilized (48 hours in a Labconco FreeZone Freeze Dry System), thoroughly mixed and up to 0.1g weighed, transferred to a 50 ml polypropylene screw-top tube and extracted once in 15ml of 70% ethanol using a Multi-Tube Pulse Vortexer (Terre Haute, IN). Extracts are then stored at -20^0^ C until assayed for hormone concentrations. Hormone concentrations are expressed per gram dry weight to control for inter-sample variation due to diet and variable moisture [38]. Wasser et al. [38] showed that expressing fecal hormones per gm dry weight controls for diet related changes in fecal bulk. Because fecal hormones are hydrophobic, removing all water from the sample removes the majority of variation in fecal bulk, significantly improving the blood-fecal hormone correspondence (see also [5] for killer whales). Samples smaller than 0.02 g dried weight were excluded from analysis to avoid inflation effects of low sample mass on hormone concentrations [39].

Radioimmunoassay was performed to measure fecal hormone metabolites using ^125^I corticosterone RIA kits (#07–120103; MP Biomedicals, Costa Mesa, CA) and MP Biomedicals' Total T3 coated tube RIA kits (#06-B254216) for GC metabolites and T3, respectively. The T3 assay was previously validated for killer whales [21]. The GC assay [36] was validated for killer whales in Ayres et al [5]. Fecal pools as well as commercial controls from each assay kit were used to assess inter-assay coefficients of variation. Commercial T3 controls were prepared as previously described [21]. P4 and T were measured using an in house 3H progesterone RIA assay using antibody CL425 [35,40], and an in-house 3^H^ testosterone RIA assay using antibody #250 [37,40]. All other hormone assays were validated in the present study.

All five hormone assays exhibited parallelism; slopes of serially diluted SRKW fecal extracts were not significantly different from the slopes of the standard curves (GC: F_1,7_ *=* 0.41, p = 0.54; T3: F_1,9_ = 2.89, p = 0.12; P4: F_1,10_ *=* 0.80, p = 0.3925; T: F_1,9_ = 3.65, p = 0.09). Fifty percent binding of the radioactively labeled hormone occurred at target dilutions of 1:60 for GC, 1:30 for T3,1:60 for P4 and 1:10 for T metabolites. All five hormones also exhibited good accuracy at their target dilutions (GC: slope = 1.2, r^2^ = 0.98; T3: 1.09, 1.00; P4: 1.07, 0.98; T: 0.68, 0.99), indicating that substances in SRKW fecal extract do not interfere with hormone binding. Inter-assay coefficients of variation were 7.8% for T3, 7.6% for GC; 17% for P4, and 19% for T. Intra-assay coefficients of variation (calculated as the percent of the mean divided by the standard deviation) were 1.9% for T3, 3% for GC, 3.1% for P4; and 3.2% for T. Antibody cross-reactivities are published in Wasser et al ([35], P4; [36], GC; [21], T3) and Velloso et al ([37], T).

### 2.4 Pregnancy assignment

All whales are photo-identified each day they are observed in the study area, making it unlikely that a newborn would be missed if present when the population is being observed [3]. This enabled us to establish temporal pregnancy profiles using fecal P4 and T concentrations for all pregnant females that subsequently gave birth, approximating gestational age at the time of sample collection based on the estimated birth date of the female’s calf. All birth dates in our study (Table 1) were estimated by two independent observers from the Center for Whale Research, respectively with 40 and 30 years experience, using close range photographs taken of each calf at the time of first observation. Features used to assess calf age included: shape of cranial crest (lumpy at birth), flopped over dorsal fin (apparent in first 1–2 days), fetal folds, fattening after first month, jaundice coloration, skin molting at 3–5 months, date of previous observed photo of pregnant females without a calf. The Center for Whale Research (unpublished data) developed these criteria by compiling a time-stamped folder of known-age calf photos that illustrate these age-dependent morphological differences.

**Table 1:** Sex, date of first observation, estimated age, birthdate and survival status for each calf whose mother was sampled during her pregnancy or lactation of that calf.

	Calf Data						Mother of Calf data		
Year	Calf ID	Calf Sex	Date Calf was first photographed	Assigned Calf Birthday	Estimated age of Calf	Calf age at death	Mother of Calf	Birth year of Mother	Age of Mother
**2007**	J42	F	5/2/2007	5/2/2007		Alive	J16	1972	35
**2008**	K42	M	6/3/2008	4/3/08	1–3 mo	Alive	K14	1977	31
**2008**	L111	F	8/12/2008	7/30/2008	2 wk	<1 month	L47	1974	34
**2009**	L112	F	2/6/2009	1/24/2009	2 wk	3 years	L86	1991	18
**2009**	J44	M	2/6/2009	1/1/2009	1 mo +	Alive	J17	1977	32
**2009**	J45	M	3/3/2009	2/15/2009	2 wk	Alive	J14	1974 (died 2016)	35
**2009**	L113	F	10/10/2009	10/1/2009	1–2 wk	Alive	L94	1995	14
**2009**	J46	F	11/11/2009	10/28/2009	2 wk	Alive	J28	1993 (died 2016)	16
**2010**	J47	M	1/3/2010	12/9/2009	< 1 mo (12/5 no calf)	Alive	J35	1998	12
**2010**	K43	F	2/21/2010	1/31/2010	3 wk	Alive	K12	1972	38
**2010**	L115	M	8/6/2010	7/31/2010	1 wk	Alive	L47	1974	36
**2010**	L116	M	10/13/2010	10/3/2010	1–2 wk	Alive	L82	1990	20
**2010**	L117	M	12/6/2010	11/30/2010	1 wk	Alive	L54	1977	33
**2010**	L114	U	2/21/2010	2/16/2010	< 1 wk	4 months	L77	1987	23
**2011**	K44	M	7/6/2011	7/3/2011	3 days (No calf 3 days prior)	Alive	K27	1994	17
**2011**	L118	F	2/10/2011	1/20/2011	3 wk?	Alive	L55	1977	34
**2011**	J48	U	2/17/2011	1/29/2011	≤ 3 wk	<1 month	J16	1972~	39
**2012**	J49	M	8/6/2012	8/6/2012	1 day, saw 1^st^ day	Alive	J37	2001	11
**2012**	L119	F	5/29/2012	5/15/2012	2 wk	Alive	L77	1987	25
**2013**	unk	U	1/7/2013	1/7/2013	1 day	<1 month	J28	1993	20
**2014**	J50	F	12/23/2014	12/15/2014	2 wk? (12/12 no calf)	Alive	J16	1972~	42
**2015**	L123	M	11/7/2015	10/15/2015	< 1 Mo (10/11 no calf)	Alive	L103	2003	12
**2015**	J53	F	10/24/2015	10/14/2015	1–2 wk (10/03 no calf)	Alive	J17	1977	38
**2015**	L122	M	9/7/2015	8/24/2015	2 wk	Alive	L91	1995	20
**2015**	J52	M	3/30/2015	3/16/2015	2 wk (no calf 02/18)	Alive	J36	1999	16
**2015**	L121	M	2/25/2015	2/18/2015	~ 1 wk	Alive	L94	1995	20
**2015**	J51	M	2/12/2015	2/5/2015	1 wk	Alive	J41	2005	10

Maternal age at time of sampling is also included.

? = best guess.

A fecal P4 concentration threshold was then established to indicate pregnancy by comparing P4 concentrations across all known sex and reproductive classes, and demonstrating that all gestating SRKW females, subsequently confirmed to have been pregnant by a live birth, surpassed this threshold and sustained it until the end of their 18 month gestation period (see also [15]). No samples from genotyped males, or from lactating, non-cycling, immature or post-reproductive females approached this P4 threshold. Comparisons of T concentrations were similarly used to separate pregnancies into early and late stages of gestation. T rises during pregnancy, albeit more slowly than P4. By mid-gestation, T concentrations in pregnant females are comparable to, if not higher than those observed only in adult males (but without a comparable rise in P4) [16] (see also results). Thus, high P4, low T samples were classified as from females in early gestation and high P4, high T samples were classified as from females in mid- to late-gestation. All samples from genotyped adult females at or above these P4 and T concentrations were classified as pregnant. Pregnancies were classified as successful if the female was subsequently observed with a live birth before 18 months from the time of sample collection. Otherwise, the pregnancies were classified as unsuccessful, representing a spontaneous abortion or an unobserved perinatal mortality.

### 2.5 Statistical analyses

All statistical analyses were performed using the software, JMP (SAS Institute, 2010). Log-transformed values were used for all hormone analyses. A general linear model (GLM) was used to distinguish reproductive and non-reproductive groups of each sex based on P4, T, T3, GC and T3/GC concentrations. Differences between groups were then tested using a chi-square contrast test.

The abundance and timing of Fraser River Chinook (FRC) was determined from 2008–2014 by Albion Test Fishery CPUE data (Catch Per Unit Effort, [41]), collected on a daily basis by an independent observer during spring, summer, and fall months. All correlations between hormone concentrations and fish abundance used Albion Test Fishery CPUE data lagged by 12 days from the time a sample was collected; the 12 day lag was derived from estimates of Chinook swim time from the study area to the test fishery, which was also in agreement with the lag time that resulted in the best fit model between prey abundance and nutritional hormones [5,8]. The CPUE data were log_10_ transformed to achieve normality. Early spring Columbia River Chinook abundance was also estimated from daily counts at the Bonneville dam [31] by calculating the area under the curve from Julian Day 100 to 140.

Vessel counts were taken every half hour (within 5 minutes of the half hour). Any vessels outside the 5 minute grace period were not counted. All boats within 0.5 mile of the killer whales were recorded by type (commercial whale watch, recreational, cargo, ferry, commercial fishing, enforcement, research, monitoring, and kayak or paddleboard) and activity (e.g., transiting, whale watching, fishing (lines in the water), acoustic, enforcing). A second (B) count was taken when a second nearby whale group was present (1–2 miles away) but outside of our initial count area, providing that the vessels and their activity could be clearly identified.

The correspondence between fish abundance and Julian date (i.e., the consecutive day of the year, ranging from 1 to 365) and vessel abundance and Julian date, across years, was established with a GLM, which allowed us to then use Julian date as proxies for fish and boat abundance in subsequent analyses. A GLM was used to separately predict T3 and GC by Julian date for all sampled individuals. The relation between early spring Columbia River salmon abundance and subsequent T3 and GC concentrations during that same year was also tested in those regressions. Finally, GLM was used to separately predict T3, GC and the T3/GC ratio, using Julian date as a polynomial and pregnancy type as independent variables. GC was included as a covariate whenever predicting T3, and vice versa, since both hormones respond to other in the regulation of energy balance. For T3, this was done by fitting T3 by GC, saving the residuals, and then using the residuals of that analysis in the final regression. For GC, the residuals for GC fit by T3 were used. In all cases, forward stepwise model selection was used to identify the best model in our GLM analyses, based on Akaike’s Information Criterion (AIC).

Raw Data are provided in S1 Appendix.

## 3. Results

In total, there were 348 samples from known (genotyped) individuals, in the final analytic dataset representing 79 unique whales (Supplemental Information-raw data), including 11 successful and 24 unsuccessful pregnancies (Table 2). Each year included a representative sampling by pod, sex and reproductive class.

**Table 2:** Pod composition and samples per unique successful and unsuccessful pregnancy from genotyped females per year.

	SRKW Pod			Reproductive Age Class				Unsuccessful Pregnancy^+^: unique whales/ total samples		Confirmed pregnancies^+^*: unique whales/ total samples	
Year	J	K	L	Juvenile	RM	RF	PRF	Low T	High T	Low T	High T
**2008**	13	5	7	7	6	7	5	0/0	0/0	1/1	1/1
**2009**	24	10	14	9	18	13	8	1/2	2/2	0/0	1/2
**2010**	14	6	12	3	6	13	10	1/1	0/0	1/2	1/1
**2011**	25	17	23	15	16	24	10	0/0	3/4	2/2	1/1
**2012**	32	11	8	6	13	24	8	5^#^/9	1^#^/2	0/0	0/0
**2013**	17	7	21	6	12	23	4	4^†^/4	1^†^/1	0/0	0/0
**2014**	36	18	6	19	10	27	4	5/6	1/1	1/4	2/2

RM = reproductive male, RF = reproductive female, PRF = Post-reproductive female.

*Not all samples between years are unique pregnancies

^†^ Includes 2 samples from one pregnancy, one with Low T and one with High T

^+^ Includes only samples from females with P4 concentrations ≥ 2000 ng/g

^#^ Observed birth, reclassified at unsuccessful due to early perinatal mortality

### 3.1 Changes in fish abundance, vessel density, T3 and GC concentrations over time

**Fig 1:**
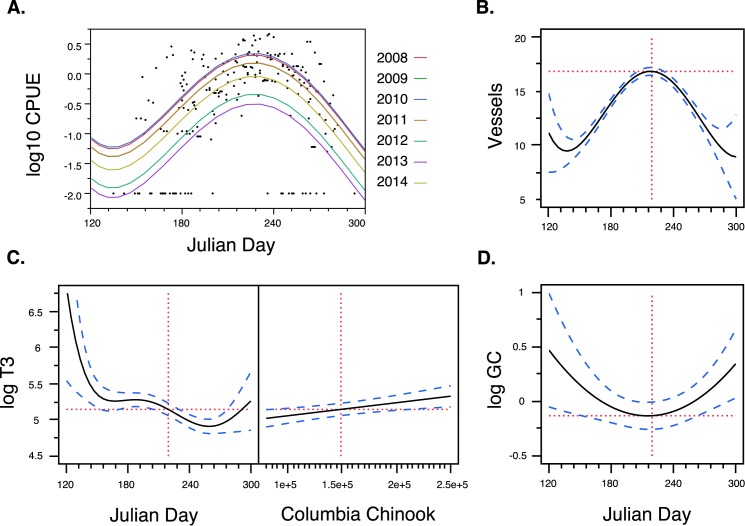
**A) Fraser River Chinook (FRC) Salmon Run abundance (CPUE: catch per unit effort), B) mean vessel count (all boats observed with 0.5 m of the whales) plotted by Julian date across years, C) Change in SRKW fecal thyroid hormone (triidothyronine, T3 ng/g dry feces) by Julian date (left panel) and early spring Columbia River Chinook abundance (right panel), and D) Change in SRKW fecal glucocorticoid (GC ng/g dry feces) hormone concentration by Julian date.** Dashed blue lines represent the standard error surrounding each curve. Vertical red line in left panel, Fig C indicates the mean peak in FRC abundance and the mean peak in boat abundance in Fig B and D.

Based on delta AIC, the Albion Test Fishery Abundance of FRC, measured in CPUE, was best predicted by a 4th order polynomial using Julian date (i.e., consecutive day of the year, P< 0.0001) across years (Fig 1A), with a peak in CPUE at day 228 (Aug 16). CPUE significantly declined across years, when examined as a continuous variable (P < 0.0001). The lowest FRC CPUE occurred in 2013, followed by 2012 (for both, p < 0.0001 compared to all prior years, and p <0.004 compared to 2014) and then 2014 (p < 0.04 compared to 2008–2011) (see also S1 Fig). Vessel density was similarly predicted by a 4th order polynomial using Julian date (p < 0.0001) with a peak at day 222 (Fig 1B). Vessel density significantly increased across years, when examined as a continuous variable (P < 0.0001).

We next separately predicted T3 and GC concentrations based on Julian date (Fig 1C and 1D, respectively), given the close association of Julian date with both fish and vessel abundance. Spring Columbia River Chinook (CRC) abundance was also included as a covariate in these analyses since the relatively slow responding T3 was hypothesized to still be influenced by spring CRC abundance at the time of SRKW early summer arrival in the Salish Sea. T3 concentration was best predicted by a 5^th^ order polynomial of Julian date (p < 0.0001) and was also positively correlated with CRC (p < 0.0001). For all years of study, T3 was at its peak when the SRKWs arrived in early summer, presumably after feeding on the early spring CRC. T3 sharply declined shortly thereafter, presumably because FRC abundance was still low, plateauing around the time that FRC CPUE begins to rise. T3 concentrations then slightly declined again in September, just after the FRC peak.

GC concentration was best predicted by the quadratic of Julian date (p = 0.004), showing the U-shaped pattern indicative of nutritional stress, with the trough at day 220, near the FRC peak. GC was not correlated with CRC, supporting the hypothesis that the GC response reflects more immediate conditions compared to T3.

### 3.2 Pregnancy occurrence and loss indices

**Fig 2:**
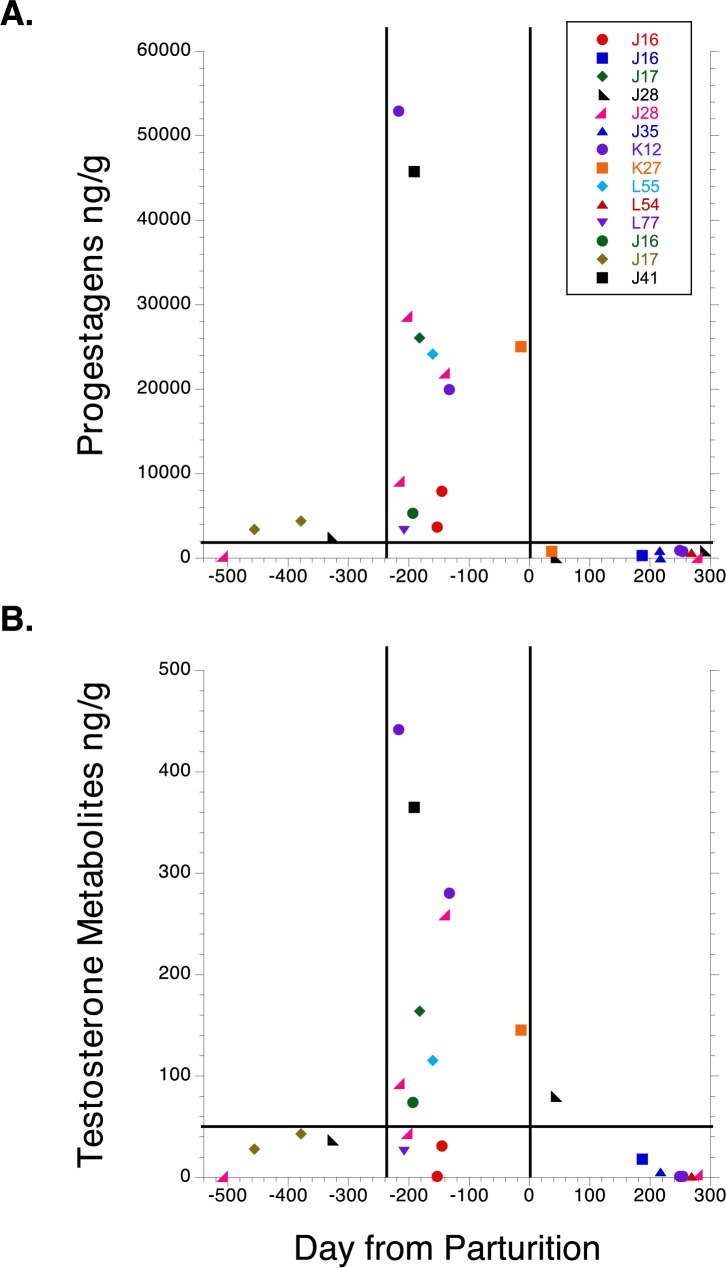
**A) Progesterone (P4) and B) testosterone (T) concentrations across gestation and lactation, for all successful pregnancies (Pg), subsequently confirmed by observed births.** Each unique pregnancy is indicated by its own symbol, along with the associated female’s ID. The vertical dashed black line in Fig A and B indicate estimated day of parturition. The 2000 ng pregnancy threshold is indicated by the horizontal dashed red line in Fig A, as is the 50 ng/g T cut-off for High and Low T samples in Fig B. The left vertical line in red indicates the Julian day where both P4 and T show sharp elevations.

Twelve females sampled during pregnancy were subsequently confirmed to give birth (37% of detected pregnancies) by photo-identification between 2008 and 2015. However, one of those females (J28) was subsequently reclassified as a High T unsuccessful pregnancy because her calf died immediately post-partum.) In all samples, P4 was well above the 2000 ng/g pregnancy threshold by 2.5 months gestation, and remained so for the next 15.5 months until parturition. One sample collected on a confirmed pregnant female during her first month of gestation had P4 levels below the 2000 ng/g threshold (Fig 2A). By contrast, no male, or immature, non-cycling, lactating or post-reproductive female whale ever approached that P4 threshold (Table 3). The majority of samples from confirmed pregnant females were well above 18,000 ng by 10 months gestation. All samples from confirmed pregnant females exhibited a precipitous decline below 2000 ng/g P4 immediately following parturition (Fig 2A).

**Table 3:** Mean hormone concentration (ng/g dry feces) and (standard error) by sex and reproductive class for each hormone measured during the study.

	Reproductive Hormones				
Sex and Reproductive Class	Thyroid (T3)	Glucocorticoid (GC)	Progesterone	Testosterone	T3/GC Ratio
**Juv F**	248.40 (40.06)	610.73 (200.17)	794.40 (268.84)*b*,*k*,*u*,*C*,*J*	3.38 (1.14)*a*,*j*,*v*,*F*	0.69 (.24)*a*,*f*
**Juv M**	229.98 (26.98)*a*,*f*	501.03 (158.82)	800.96 (73.99)*a*,*j*,*t*,*B*,*K*,*O*	30.11 (7.84)*a-i*	0.44 (.05)*b*,*f*
**Pub F**	264.19 (47.49)*d*,*i*	955.08 (286.02)	305.90 (95.0)*g*,*q*,*y*,*F*,*H*,*J-N*	3.80 (1.90)*h*,*p*,*y*,*D*,*H*	0.70 (.31)*d*
**Pub M**	230.99 (29.34)*e*	1244.21 (310.87)	258.11 (42.15)*h*,*r*,*z*,*G*,*I*,*O-R*	19.32 (6.08)*q*,*A-E*	0.71 (.35)
**Ad M**	167.07 (10.63)*a-e*	1073.14 (114.92)	579.57 (38.14)*I*,*s*,*H-I*	126.67 (17.73)*I*,*r*,*u*,*w*,*z*,*E-H*	0.32 (.044)*e*,*f*
**Ad F no-calf**	169.97 (14.13)	1004.21 (135.15)	651.83 (68.28)*d*,*m*,*w*,*A*,*D*,*M*,*Q*	5.12 (1.60)*c*,*l*,*x*,*B*	0.35 (.057)
**LoT Conf**	250.78 (35.63)*c*,*h*	1127.81 (233.66)	6205.89 (2564.93)*g*,*o*,*B-G*	21.28 (5.78)*n*,*x-z*	0.37 (.14)
**LoT Upg**	252.56 (27.06)*b*,*g*,*i*	1288.23 (228.05)	6618.20 (2014.13)*e*,*n*,*t-z*,A	11.32 (3.2)*e*,*m*,*s-u*	0.82 (0.46)
**HiT Conf**	218.05 (45.6)	1057.31 (477.75)*a*	25587.17 (5116.49)*a-i*	215.34 (42.87)*f*,*t*,*v*,*w*	1.11 (.42)*c*,*e*
**HiT Upg**	177.1 (26.98)	1787.20 (467.83)*a*	37425.73 (12819.62)*j-s*	197.95 (39.7)*d*,*j-r*	0.16 (.035)*a-d*
**Lactating**	165.02 (24.70)*f-i*	1094.36 (270.03)	650.12 (84.68)*c*,*l*,*v*,*C*,*L*,*P*	22.71 (13.33)*b*,*k*,*s*,*A*,*G*	2.05 (1.59)
**Post-Reprod F**	199.01 (19.82)*j*	1039.2 (133.11)	662.30 (66.62)*f*,*p*,*x*,*y*,*E*,*N*,*R*	7.88 (1.89)*c*,*o*,*C*	0.36 (.068)

Significant differences between means in any two cells within the same column are indicated by the same italicized letter in both cells.

F = female, M = male, Juv = juvenile; Pub = pubescent, Ad = adult, T = testosterone, Conf = confirmed pregnant female by subsequent observation of a live calf; UPg = unsuccessful pregnancy.

T concentrations of all samples from confirmed pregnant females clearly remained below 50 ng/g until mid-gestation (Fig 2B). Thus, pregnancy samples (i.e., samples above the 2000 ng/g P4 threshold) were divided into low (≤ 50 ng/g) and high (> 50 ng/g) T groups, respectively, corresponding to early, and mid-to-late stages of gestation (Fig 2A and 2B). The only other age-sex class that showed significantly elevated T concentrations, above the 50 ng/g threshold, was adult males, but their P4 concentrations never approached 2000 ng/g (see Table 3). T was above the 20 ng/g by 2.5 months gestation in all confirmed pregnant females, with the majority above 100 ng/g by 10 months gestation (Fig 2B). Low T confirmed pregnant females had a mean fecal P4 of 6206 ng/g ± 2565) and a mean T concentration of 21 ng/g ± 5.8, whereas High T confirmed pregnant females had a mean fecal P4 > 25587 ng/g ± 5116) and a mean T concentration of 215 ng/g ± 43 (Table 3). With the exception of one early lactation sample, testosterone concentrations declined well below the 50 ng/g threshold after parturition (Fig 2B). Multiple scat samples were obtained from the same pregnancy event in 4 of the 11 pregnancies and three lactation events; all multiple samples exhibited these same P4 and T patterns over time.

None of the post-reproductive females were ever recorded to be pregnant nor did they show any sign of ovarian activity (Table 3). These results support the assertion that the “post-reproductive” adult females (>40 years of age) in this population have undergone reproductive senescence [42].

**Fig 3:**
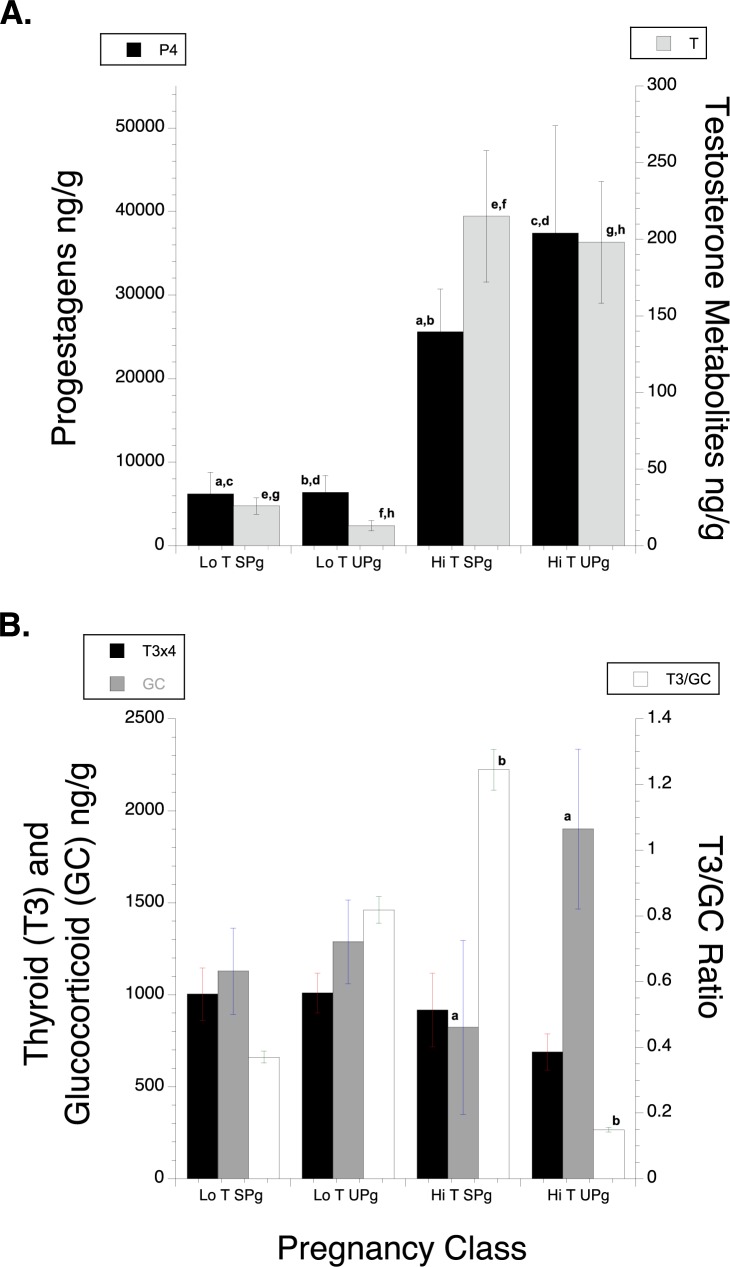
**A) Mean P4 and T concentrations and B) mean tri-iodothyronine (T3) and glucocorticoid (GC) concentrations, along with the T3/GC ratio, for Low and High T successful (SPg) and unsuccessful pregnancies (UPg).** Corresponding values for all sex and reproductive classes of SRKWs, including significant differences between classes, are presented in Table 3. Note: T3 Concentrations are multiplied by 4 in Fig B to scale its concentrations to those of GC in order to present a double Y graph for 3 related metrics, each with different value ranges. Bars with the same letter are significantly different from each other.

Samples from genotyped reproductive age adult females with P4 concentrations above the 2000 ng/g pregnancy threshold that were not followed by a live calf within the 18-month gestation period were assumed to be from females that experienced a spontaneous abortion (in utero mortality), or early perinatal death prior to calf’s first observation, collectively termed an unsuccessful pregnancy (UPg). Among the females classified as reproductive adults, we characterized 24 unique unsuccessful pregnancy (UPg) events from 12 different females with genotyped samples collected between 2008–2014—up to 69% of all confirmed pregnancies (Table 2). All samples from the 22 apparent UPg’s had significantly elevated progesterone concentrations well above 2000 ng/g. Yet, no observations of those females over the next 18 months included a new calf. As with confirmed pregnancies, the presumed UPg samples were separated into two distinct groups: one with T concentrations above 50 ng/g feces (mean T = 198.6±40; P4 = 37,425±12,820), hereafter termed “high T UPg” samples (7 unique females, 7 presumed late spontaneous abortions and one early perinatal loss), and the other with T concentrations below 50 ng/g feces (mean T = 11.3±3.2; P4 = 6618±2014), termed “low T UPg” samples (4 females, 16 presumed early spontaneous abortions; Table 2; Fig 3A). Multiple samples from 6 of the 24 unsuccessful pregnancy samples (4 low T, 2 high T, plus 1 low T that transitioned to high T) were all within the pregnancy range (i.e., P4 < 2000 ng/g). Thirty three percent of the UPg samples (8 out of 24) identified here were high T UPg (up to 23% of all recorded pregnancies). The high T UPg samples were likely from the second half of gestation, based on their high P4 and T concentrations relative to temporal profiles for those hormones in whales with a confirmed pregnancy (see Fig 2).

T3 and GC concentrations also varied across all sex, age and reproductive classes (Table 3). T3 was highest in juvenile and pubescent individuals compared to adults, with the exception of Low and High T successful pregnant and low T UPg females. All of those individuals also had a relatively high T3/GC ratio (> 0.3), indicative of relatively good nutrition (Table 3). By contrast, T3 in the High T UPg samples was comparable to that of non-pregnant adults (Table 3), and notably lower than the concentrations from successful pregnant and low T UPg females (Fig 3B). These High T UPg samples also had the highest GC concentrations of any reproductive class, was significantly higher than the GC concentrations in High T successful pregnancies. The T3/GC ratio in High T UPg females was lower than that of another other reproductive class (Table 3), indicative of nutritional stress (Table 3), and nearly 7 times lower than that among High T successful pregnancies. Indeed, the T3/GC ratio in High T successful pregnancies was higher than that for any other reproductive class, with the exception of lactating females (Table 3, Fig 3B).

### 3.3 Changes in T3 and GC concentrations relative to fish abundance over time across pregnancy groups

**Fig 4:**
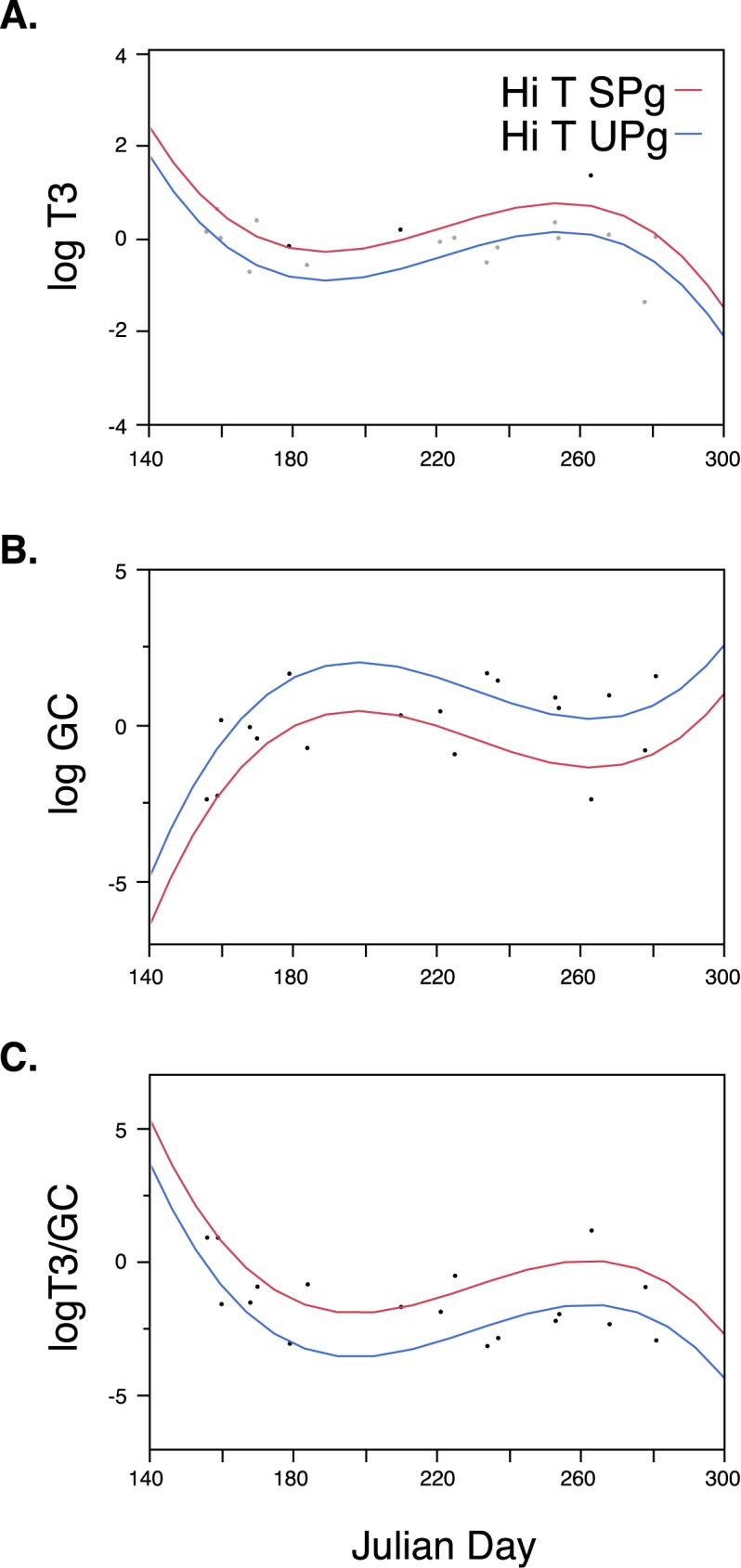
A) T3 and B) GC concentrations, along with (C) the T3/GC ratio, by Julian day for High T successful pregnancies (SPg) versus High T unsuccessful pregnancies (UPg).

T3 and GC concentrations, along with the T3/GC ratios were separately compared among High T successful pregnant and UPg samples, across Julian date. (Low T samples were not included in these comparisons because their T3 and GC concentrations were not significantly different from those of confirmed pregnant females.) All three dependent variables were best predicted by a 3^rd^ order polynomial of Julian date (p < 0.01). Similar to the overall population trend, T3 concentrations were highest in early summer, followed by a precipitous decline. However, the initial T3 decline was longer in duration than that observed for the overall population, lasting until day 190. T3 concentrations in the pregnant females then increased until day 250 (Fig 4A), which was near the time when the FRC run reached it back (Fig 1A). While the pattern was the same in High T successful and unsuccessful pregnancies, T3 in High T UPg samples remained significantly lower than that in High T successful pregnant females (p = 0.004), consistent with relatively higher nutritional stress in the High T UPg females (Fig 4A). Change in GC concentrations among pregnancy females were the exact opposite of T3, showing a steep rise until day 190 followed by a decline until day 250, and significantly higher in High T UPg compared to High T successfully pregnant females (p < 0.002) throughout this period (Fig 4B). Change in the T3/GC ratio followed the same pattern as T3, also remaining significantly higher in HighT successful pregnancies (p< 0.003) (Fig 4C).

## 4. Discussion

Reproductive failure in response to conditions that jeopardize offspring survival has been described as an adaptive response if conditions are likely to improve in the foreseeable future. This environmentally-mediated loss most commonly occurs early in reproduction (conception and early pregnancy) when the cost of suppression (e.g., lost time and energy; impacts on maternal health) is relatively low [43,44]. However, failure at later stages of reproduction is expected when cues indicating poor fetal or neonatal conditions present themselves late in the reproductive event. The longer the span between conception and birth the more likely later suppression is to occur. Premature birth is a relatively low risk way to suppress reproduction because the reproductive failure occurs post-partum with reduced chance of infection. However, its occurrence should still depend on when harsh conditions present themselves. If fetal demise occurs or environmental conditions become especially harsh (e.g., risk of sepsis from starvation induced ketoacidosis during pregnancy; [45]), spontaneous abortion is expected. Thus, spontaneous abortion, premature birth, still birth, and perinatal and neonatal mortality are all part of a continuum of reproductive suppression that present with harsh conditions, on balance with risk of reproductive loss at that stage of reproduction [44,46].

SRKWs have an 18 month gestation period and their nutritional health depends on the relative timing of multiple, seasonal fish runs (e.g., spring CRC and summer FRC), as well as food availability in between those periods, each of which vary markedly between years (S1 Fig). The increasingly common occurrence of SRKW births outside the typical winter calving period may well be an indication of the increased unpredictability of diminishing fish runs along with the corresponding high rate of late reproductive loss in SRKWs, including more costly late spontaneous abortions. The SRKWs had a 69% pregnancy failure rate during our study and an unprecedented half of those occurred at later stages of reproduction when the energetic cost of failure and physiological risk to the mother was relatively high. Temporal patterns in T3 and GC hormone profiles suggest that the SRKWs are experiencing periodic nutritional stress, partly caused by variation in the relative timing and strength of seasonal FRC and CRC runs (Fig 1). This nutritional stress is significantly associated with unsuccessful pregnancies in SRKWs (Figs 3 and 4), impairing the potential for population recovery through low recruitment as well as risk to the health and survival of the limited number of reproductive-age females.

High T (mid-to-late gestation) females with successful pregnancies in our study had significantly higher T3 and lower GC concentrations, as well as a substantially higher T3/GC ratio over time, compared to High T unsuccessful pregnancies (Figs 3 and 4). This indicates that successfully pregnant females arrived in the Salish Sea in significantly better nutritional condition, and remained so compared to UPg females that experienced pregnancy loss some time after mid-pregnancy. West et al [25] similarly found significantly higher total T3 concentrations among adult females in successful compared to unsuccessful pregnancies at all stages of gestation among captive dolphins.

Only 4 detected pregnancies between 2011–2013 resulted in live births when Fraser River Chinook and early spring Columbia River Chinook runs were both exceedingly low. Just one of those births occurred in 2013, when both FRC and CRC abundances were at their lowest, and that animal died almost immediately post-partum. By contrast, there were up to 9 early gestation (Low T) and 5 mid to late gestation (High T) unsuccessful pregnancies detected during that same 3 year period, with almost half of these early-term and one of the mid to late term unsuccessful pregnancies occurring in 2013. That trend reversed in 2014, with relatively high CRC returns and early onset of FRC returns in 2014 and 2015 (S1 Fig, Appendix) that was followed by 8 new births between December of 2014 and October 2015; however, up to 6 unsuccessful pregnancies still occurred that year, five of which occurred early in gestation (Low T Upg).

High T UPg samples were either from late spontaneous abortions (also known as intrauterine fetal demise), or undocumented perinatal or neonatal deaths where the infant disappeared prior to first observation. The lack of observed perinatal or neonatal deaths when most successful births during our study were observed within 2 weeks of parturition (Table 1), led us to estimate that a substantial portion of the High T UPg samples represented late spontaneous abortions. Although the negative effect of these later reproductive losses on SRKW population growth is roughly the same, infection from a failed or incomplete abortion likely poses a greater risk of removing a reproductive female from the breeding population. At least one SRKW stranding was confirmed to be a pregnant female with infection from a retained fetus listed as the cause of maternal death (J32, December 2014).

Reproductive loss among women during the well-documented 1945 Dutch Famine may exemplify the kinds of impacts expected in response to severe nutritional stress among SRKWs, since: both humans and SRKWs have relatively long interbirth intervals (gestation length and extended lactation amenorrhea), starvation was acute and the Dutch Famine outcomes were not biased by interventions from modern health care [44,47,48]. The Nazis closed off the borders of Holland between October 1944 and May 1945, causing massive starvation over a 5–8 month period, with good food conditions before and after. There was a one-third decline in the expected number of births among confirmed pregnant woman during the under-nutrition period. Conceptions during the hunger period were very low. However, women who conceived during the hunger period had higher rates of abortion, premature and stillbirths, neonatal mortality and malformation. Nutrition had its greatest impact on birth weight and length for mothers experiencing hunger during their second half of gestation, when the fetus is growing most rapidly [47].

Many of the unsuccessful pregnancies in our study were based on single genotyped samples, and it is possible that pregnancy failure rates could be somewhat overestimated. For example, we cannot rule out that some portion of the singleton Low T samples were actually from post-ovulatory luteal phase females that did not produce a detectable conception. Some low T samples could also be from pseudo-pregnancies, although those are rare, have only been reported in captivity [49], and could be an artifact of captive husbandry where males and females are housed separately. It is unlikely that any post-ovulatory luteal phase samples were misclassified as High T UPg samples because both P4 and T concentrations in the High T samples were all well above those expected for luteal phase samples (Table 3, Fig 2). Moreover, Robeck et al [15,16] clearly distinguished luteal phase samples from pregnant samples by 4 weeks of gestation. This is consistent with our findings from Fig 2, indicating pregnancy detection among females by 100 days of gestation. Given the above, we consider only a small portion of the 8 singleton, low T UPg samples with P4 above the 2000 ng/g pregnancy threshold to be possibly misclassified as early abortions. However, the consistency of these patterns on multiple endocrine and temporal measures, across years, strengthens the assertion that pregnancy failure is a major constraint on killer whale population growth, triggered by insufficient prey.

The rise in fecal P4 concentrations that we observed among successful pregnancies was somewhat delayed compared to that observed in serum from captive killer whales [15]. This could suggest that our estimated birth dates, and hence our projected conception dates, actually occurred earlier than expected, increasing the likelihood that some perinatal mortalities were misclassified as late spontaneous abortions. However, the delayed P4 peak in feces of pregnant SRKWs compared to Robeck et al [15] most likely resulted from differences in the P4 metabolites measured in feces versus serum. The predominant P4 metabolite measured by our antibody is 5α-DHP [35]. Using an EIA version of the P4 antibody we used in our study, Robeck et al [15] found that 5α -DHP did not become the predominant progesterone metabolite in captive killer whale serum until 161–360 days of gestation, and remained secondarily so from 361 days gestation to term. Fecal progesterone metabolites spiked around mid-pregnancy in our study, consistent with the time when 5α -DHP predominated in serum [15]. It is also noteworthy that our testosterone antibody [37,40] followed a similar temporal pattern in SRKW to that described for captive whales by [16]. That also supports the reliability of our projected conception dates and occurrences of spontaneous abortion.

Exposure to persistent organic pollutants (POPs)—lipophilic compounds with established adverse health effects—in response to food stress add yet another cumulative risk of fetal demise and/or perinatal and neonatal mortality. Lundin et al. [8,50] showed that POPs, namely PCBs, DDTs, and PBDEs, increase in circulation in SRKWs when Fraser River Chinook abundance is lowest, presumably due to increased fat metabolism in response to nutritional stress. Mobilization of contaminants into circulation also occurs during the energetic demands of lactation, with an estimated 70–90% lactation transfer of maternal toxicant burden in primiparous females [51]. High POP burden has specifically been associated with disruption of reproduction success and reduced calf survival in marine mammals [52–55]. Most notably, Lundin et al. [8] found increased Persistent PCBs, the group of PCBs considered more persistent and more toxic [56], in the female whales classified with UPg’s (73%; 95% CI, 61–85) compared to all other female reproductive groups (range 43–56%). Further evidence in support of the occurrence of UPg in this population is the unexpected inverse in bioaccumulation of POPs with age in “nulliparous” mature females (3 of 4 nulliparous whales had an unsuccessful pregnancy defined by fecal hormone measures). This occurrence is likely explained by toxicant offloading from an undocumented pregnancy or neonate loss.

Both poor nutrition and increased POP loads have each been demonstrated to suppress T3, which negatively impacts fetal brain growth [22,57,58]; immunosuppression may also occur, increasing risk of infection [53,59–61]. Salmon are the Southern Resident killer whales predominant prey and main source of toxic exposures [62,63]. This relation of reduced food supply and increased exposure to lipophilic POPs could be similarly impacting coastal Native American communities that depend on this same seasonal salmon resource and also appear to be experiencing high rates of reproductive loss [64,65].

Results of the SRKW study strongly suggest that recovering Fraser River (FRC) and Columbia River Chinook (CRC) runs should be among the highest priorities for managers aiming to recover this endangered population of killer whales. SRKW are suffering significant reproductive loss due to lack of Chinook prey and associated effects (e.g., release of lipophilic toxins into circulation). The FRC run is a major prey source for the SRKW population during summer and early fall, and appears to be key to providing the needed reserves to carry the whales through the subsequent winter [6]. The early spring CRC runs likely serve to replenish energetic reserves expended during the previous winter as well as help sustain the whales until the occurrence of the subsequent late summer peak in the FRC runs. The relative importance of the early spring Columbia River Chinook run likely became all the more critical to the SRKWs as historic FRC runs that peaked earlier in summer became depleted from overfishing and habitat destruction [6]. Other species, including people, also appear to be impacted by these conditions.

Without steps taken to remedy the situation, we risk losing the endangered SRKW, an extraordinarily important and iconic species to the Pacific Northwest. Since strengthening relevant Chinook runs should significantly decrease physiological stress and increase pregnancy success rates in SRKW during the same year that fish runs increase, the physiological indices used in this study could also provide rapid assessment tools for guiding adaptive management of SRKW populations. Historical and modern dependence on fish as an essential food source for coastal communities with limited resources, in conjunction with growing food shortages and increased risk of toxicant exposure, has international implications.

## Supporting information

S1 Fig Timing and abundance of Columbia River (orange) and Fraser River (blue) Chinook runs based on DART (2015) and Albion Test fisheries (Catch Per Unit Effort, Albion 2015), respectively (see also Lundin 2015). (TIFF)

S1 Appendix Raw data. (XLSX)
