# Supplementary figures and images for: Population growth is limited by nutritional impacts on pregnancy success in endangered Southern Resident killer whales (Orcinus orca)
Source: PLoS One. 2017 Jun 29;12(6):e0179824. doi: 10.1371/journal.pone.0179824 (PMC5491047; doi:10.1371/journal.pone.0179824)

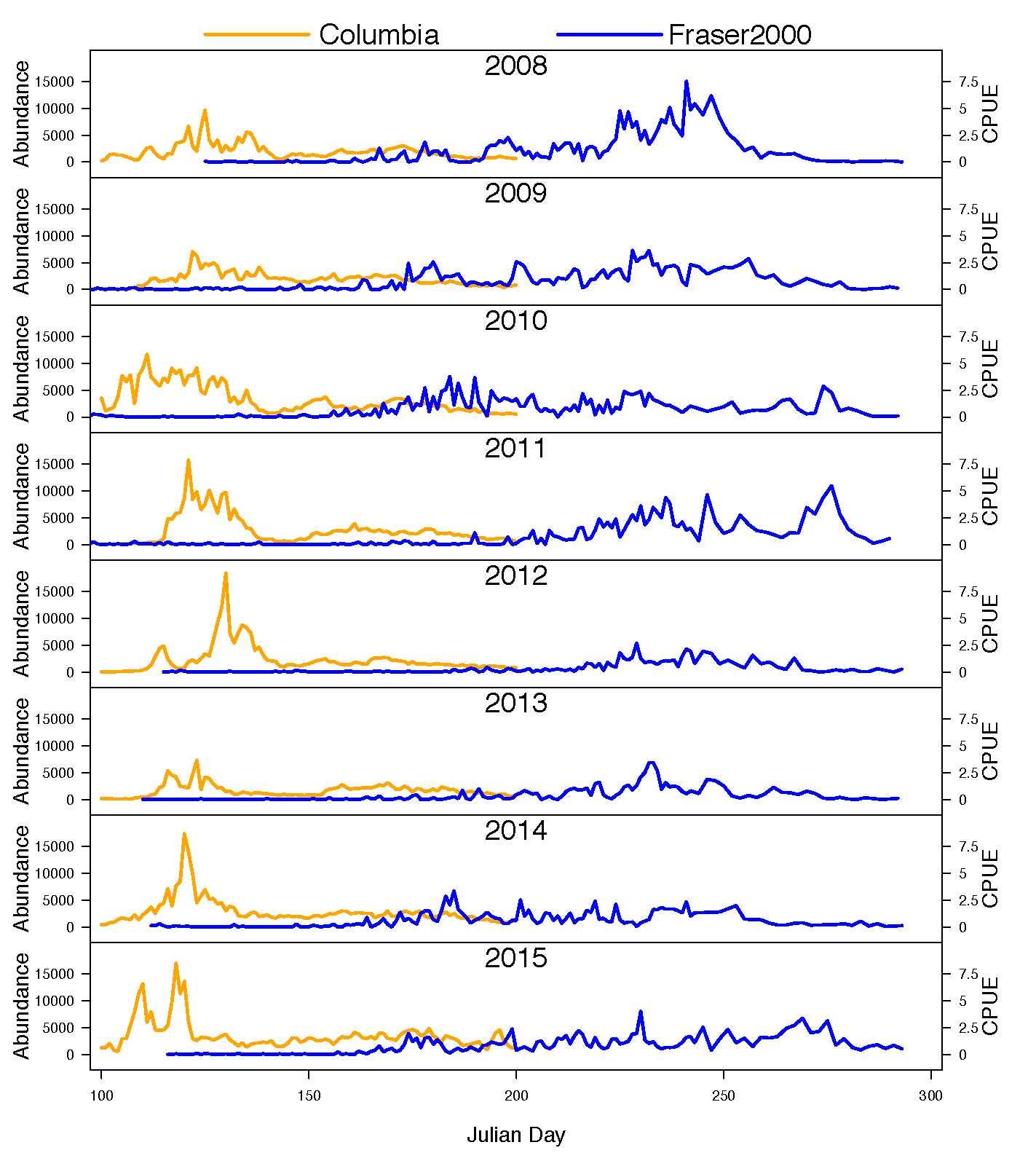

Supplement: S1 Fig — (TIFF) [file pone.0179824.s001.tiff]
